# Activity-Dependent Subcellular Cotrafficking of the Small GTPase Rem2 and Ca2+/CaM-Dependent Protein Kinase IIα

**DOI:** 10.1371/journal.pone.0041185

**Published:** 2012-07-18

**Authors:** Robyn Flynn, Etienne Labrie-Dion, Nikolas Bernier, Michael A. Colicos, Paul De Koninck, Gerald W. Zamponi

**Affiliations:** 1 Department of Physiology and Pharmacology, Hotchkiss Brain Institute, University of Calgary, Calgary, Alberta, Canada; 2 Centre de Recherche de l’Institut universitaire en santé mentale de Québec, Québec City, Québec, Canada; 3 Département de Biochimie, Microbiologie et Bio-Informatique, Université Laval, Québec City, Québec, Canada; Vanderbilt University Medical Center, United States of America

## Abstract

**Background:**

Rem2 is a small monomeric GTP-binding protein of the RGK family, whose known functions are modulation of calcium channel currents and alterations of cytoskeletal architecture. Rem2 is the only RGK protein found predominantly in the brain, where it has been linked to synaptic development. We wished to determine the effect of neuronal activity on the subcellular distribution of Rem2 and its interacting partners.

**Results:**

We show that Rem2 undergoes activity-and N-Methyl-D-Aspartate Receptor (NMDAR)-dependent translocation in rat hippocampal neurons. This redistribution of Rem2, from a diffuse pattern to one that is highly punctate, is dependent on Ca^2+^ influx, on binding to calmodulin (CaM), and also involves an auto-inhibitory domain within the Rem2 distal C-terminus region. We found that Rem2 can bind to Ca^2+^/CaM-dependent protein kinase IIα (CaMKII) a in Ca^2+^/CaM-dependent manner. Furthermore, our data reveal a spatial and temporal correlation between NMDAR-dependent clustering of Rem2 and CaMKII in neurons, indicating co-assembly and co-trafficking in neurons. Finally, we show that inhibiting CaMKII aggregation in neurons and HEK cells reduces Rem2 clustering, and that Rem2 affects the baseline distribution of CaMKII in HEK cells.

**Conclusions:**

Our data suggest a novel function for Rem2 in co-trafficking with CaMKII, and thus potentially expose a role in neuronal plasticity.

## Introduction

Activity-dependent remodelling of neurons is a key contributor to long-term plasticity in the nervous system. Neuronal stimulation activates a number of Ca^2+^-dependent cell signaling processes that lead to rearrangements of the cytoskeleton, thereby causing neurons to extend or retract processes, and to alter synaptic strength (reviewed in [1]). One of the Ca^2+^ dependent enzymes involved in neuronal plasticity is calmodulin (CaM)-dependent protein kinase II (CaMKII). Upon strong neuronal activation, CaMKII undergoes a rapid redistribution from a diffuse to a punctate pattern [2]. This form of aggregation, also termed self-association, is thought to involve an interaction between the catalytic and regulatory domains of individual subunits from separate CaMKII multimers. Since each CaMKII multimer has 12 subunits, these interactions can thus lead to the aggregation of several multimers together [2]. This process may support the recruitment of CaMKII to post-synaptic sites after the activation of the N-Methyl-D-Aspartate receptors (NMDARs) [2], consistent with the “tower-like” structures emerging from post-synaptic densities, which have been observed by immuno-electron microscopy. The multivalent nature of CaMKII and its ability to bind a very wide range of proteins suggest that its dynamic, activity-dependent translocation in active neurons could i) be regulated by interacting structural or signaling proteins and/or ii) serve to recruit together these proteins within the CaMKII scaffolds at strategic sites such as the synapse or intra-somatic elements.

One possible regulator of CaMKII action is the RGK (Rad, Gem/Kir) family of Ras-related small GTPases, which includes the proteins Rad, Gem/Kir, Rem and Rem2 (reviewed in [3]). Although commonly considered to be important regulators of high voltage activated Ca^2+^ channels [4]–[7], they are known to be involved in cytoskeletal rearrangement [8], [9]. The small GTPase Rad, which is expressed predominantly in heart and muscle, has been shown to bind to CaM and to immunoprecipitate with CaMKII [10]. The neuronal homolog of Rad, Rem2 [11] also interacts with CaM [7], and furthermore has been shown to regulate dendritic morphology in a CaM-dependent manner [12]. Given that Rem2 and CaMKII both interact with CaM and with cytoskeletal elements [13], and that both proteins regulate spine size [12], [14], we hypothesized that Rem2 and CaMKII interact with each other, and thereby co-influence their subcellular trafficking in neurons upon changes in neuronal activity. Indeed, we show here that Rem2 interacts with CaMKII, and in doing so, alters the subcellular localization of CaMKII. Stimulation of hippocampal neurons mediates an NMDA-and Ca^2+^/CaM-dependent dynamic redistribution of Rem2 into clusters, which correlated spatially and temporally with clustering of CaMKII. Finally, we show that CaMKII clustering is required for that of Rem2. Our results then indicate interdependent roles of both proteins in subcellular trafficking and thus potentially in neuronal plasticity.

## Results

### Rem2 Redistributes in Response to Neuronal Stimulation

**Figure 1:**
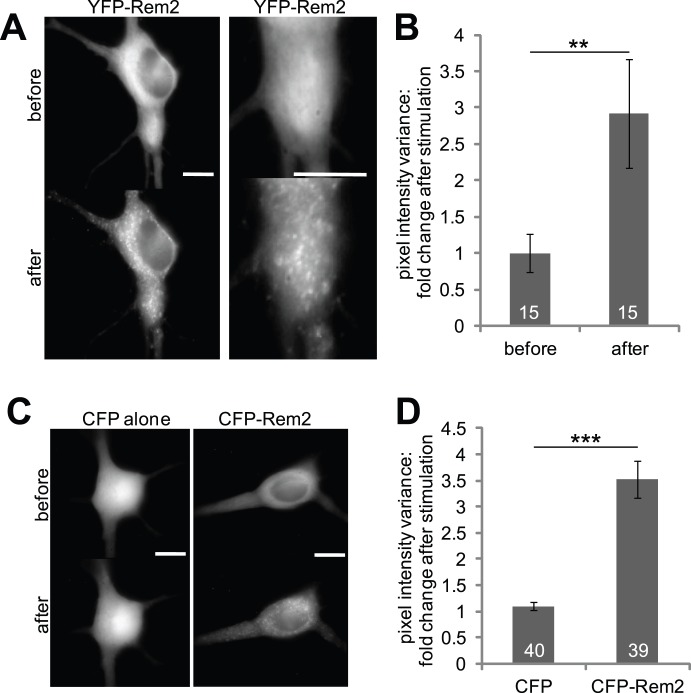
Rem2 overexpressed in neurons redistributes in response to neuronal stimulation. (**A**) Images of neurons expressing either YFP or YFP-Rem2, before and after photoconductive stimulation, a non-invasive method of stimulating individual cells in a culture using light to target neurons grown on a silicon chip. To stimulate specific cells, we targeted the cell bodies of transfected neurons using a YFP filter, then stimulated the chip using 3–5 V of current for 2 msec duration at 20 Hz for 5 sec. Cells were imaged before and after stimulation to compare Rem2 distribution. Right panel is a magnification of the left panel to show details of Rem2 puncta. Scale bars represent 10 µm. (**B**) Normalized pixel value variance before stimulation, 1.0±0.26; after stimulation, 2.92±0.75, N = 15; paired t-test p = 0.006. Error bars represent SEM, and numbers within the bars indicate the number of cells averaged. (**C**) Images of neurons expressing either CFP or CFP-Rem2, before and after chemical stimulation. Individual cells in a culture of dissociated hippocampal neurons were imaged and their positions recorded, then the culture was stimulated with bath application of 25–100 µM glutamate/2.5–10 µM glycine. Follow-up images were taken of the recorded cells within 7 minutes of stimulation. Scale bars represent 10 µm. (**D**) Images such as in panel C were filtered and quantified by the variance in pixel values in each image. Variance was normalized to unstimulated cells. Ratio of CFP alone after/before stimulation, 1.10±0.07, N = 40; ratio of CFP-Rem2, 3.52±0.36, N = 39; t-test p<0.001. Numbers within the bars denote numbers of cells averaged for each bar. Error bars are SEM.

To investigate the spatial dynamics of Rem2 in neurons, we created a series of fluorescent protein-tagged Rem2 constructs and expressed them in cultured rat hippocampal neurons. In the absence of stimulation, neurons with YFP-Rem2 displayed a diffuse distribution of fluorescence. Following photoconductive stimulation, a non-invasive technique that uses focused light to depolarize individual neurons in cultures grown on silicon wafers [15], YFP-Rem2 fluorescence became redistributed from a diffuse to a punctate distribution (Figure 1A and B). A similar redistribution of the CFP-Rem2 signal occurred when neurons were stimulated by application of glutamate/glycine, whereas unconjugated CFP did not show any change in subcellular distribution after stimulation (Figure 1C & D). To ensure that the redistribution of Rem2 was not due to its fusion to a large CFP fluorophore, we conducted similar experiments using HA-Rem2. As shown in Figure S1, puncta of HA-Rem2 overlapped with those of GFP-Rem2, indicating that the fluorescent tag does not contribute to Rem2 redistribution. To ensure that Rem2 aggregation was not due to loss of calcium homeostasis or impending cell death during neuronal stimulation, we stained stimulated cells expressing GFP-Rem2 with propidium iodide. Zero out of 20 Rem2-expressing cells showed propidium iodide staining before stimulation, and only 1/26 showed staining after stimulation. This indicates that Rem2 aggregation does not occur as a result of early cell death pathways. Overall, these data indicate that neuronal activity and activation of glutamate receptors cause a robust change in the subcellular distribution of Rem2. Given that photoconductive stimulation and glutamate receptor activation mediated virtually identical effects, ensuing experiments were conducted with the chemical stimulation protocol for simplicity.

### Rem2 Redistribution Depends on Calcium Influx via NMDA Receptors

**Figure 2:**
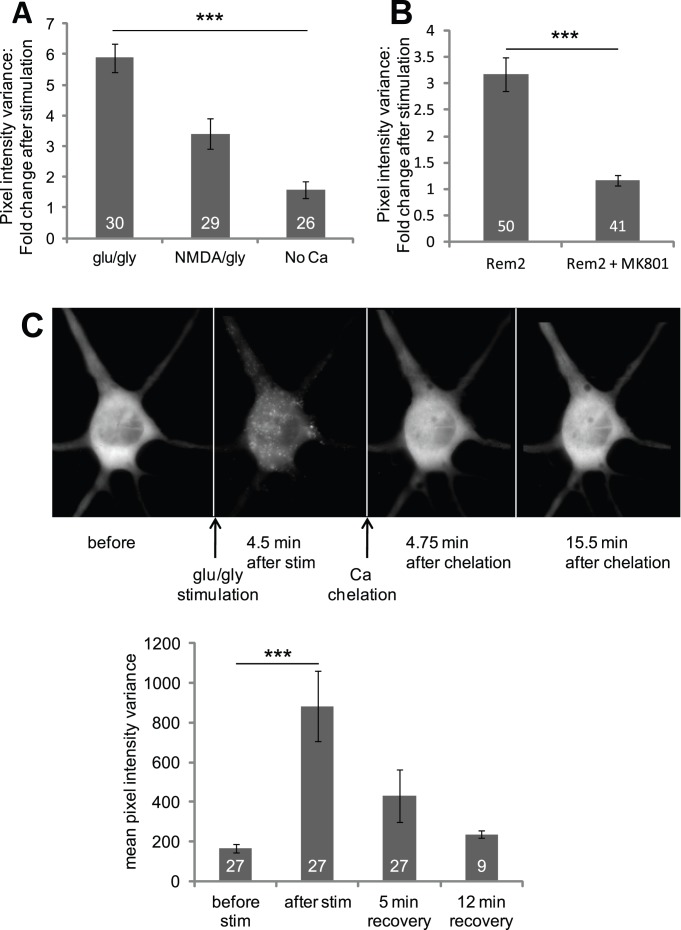
Rem2 redistribution is dependent on calcium influx via the activation of NMDA receptors. (**A**) Neuronal cultures were stimulated for 60 seconds with either 100 µM glutamate/10 µM glycine (glu/gly and No Ca conditions) or 100 µM NMDA/10 µM glycine. In the No Ca condition, neurons were incubated in EBS without 3 mM CaCl_2_, in the presence of 30 nM TTX to reduce spontaneous activity, then stimulated as above. Ratio after/before of Rem2 stimulated with glutamate/glycine, 5.88±0.47, N = 30; with NMDA/glycine, 3.41±0.49, N = 29, one-way ANOVA followed by Bonferroni post-hoc test p<0.005 compared to glutamate/glycine; no calcium + TTX, 1.58±0.28, N = 26, p<0.001 compared to glutamate/glycine. Numbers within the bars denote N, and error bars represent SEM. (**B**) Redistribution of Rem2 is blocked by MK-801. Neuronal cultures were treated with the NMDAR open-pore blocker MK-801 (62.5 µM) for 20 minutes, and then stimulated for 60 seconds with glutamate/glycine. Controls experienced the same stimulation protocol in the absence of MK-801. This treatment abolished redistribution (ratio after/before of Rem2 alone, 3.17±0.31, N = 50; Rem2+ MK-801, 1.16±0.10, N = 41; t-test p<0.001). (**C**) Rem2 redistribution is reversed by removing calcium. (Upper) Serial images of individual cells were taken before and after stimulation for 60 seconds with 100 µM glutamate/10 µM glycine. 5 minutes after stimulation, calcium was removed by replacing the bath solution with chelation solution lacking calcium and containing 1 mM EGTA. Images were taken about 5 minutes and 15 minutes after incubating the neurons in chelation solution. (Lower) Quantitation of Rem2 redistribution reversal. Pixel value variance is significantly different after stimulation (882±177 after vs. 164±21 before, one-way repeated measures ANOVA followed by Dunnett’s post-hoc test: N = 27 for each, p<0.001), but goes back to baseline level following calcium chelation (5 min after chelation, 430±130, N = 27; 12 min after chelation, 237±17, N = 9; difference from before stimulation is NS).

The observation that the application of glutamate/glycine mimicked the effects of photoconductive stimulation suggests an involvement of NMDARs. Indeed, Rem2 underwent redistribution in response to application of NMDA/glycine, albeit to a somewhat lesser extent than that of glutamate/glycine (Figure 2A), thus implicating NMDARs. NMDARs are a major source of Ca^2+^ influx into neurons. To test whether Ca^2+^ entry via these receptors was necessary, we examined Rem2 redistribution in the presence and the absence of extracellular Ca^2+^. As shown in Figure 2A, cells stimulated with glutamate/glycine in a Ca^2+^-free extracellular bath solution showed minimal Rem2 redistribution. This suggests that Ca^2+^ is essential for redistribution, and that some Ca^2+^ must first enter the cell to trigger this process. Finally, redistribution was completely blocked by addition of the specific NMDAR pore-blocker MK-801 (Figure 2B), showing that Ca^2+^ must first enter the cell specifically through the NMDAR to induce Rem2 redistribution.

The redistribution of Rem2 following glutamate/glycine stimulation was only partially reversible after several minutes of washout with control solution (data not shown). However, when the external bath solution was replaced by one containing 1 mM EGTA and zero Ca^2+^, Rem2 puncta elicited by glutamate/glycine stimulation were quickly dispersed, and the distribution of Rem2 returned to a pre-stimulation state (Figure 2C). These data suggest that this Rem2 aggregation can be reversed, but that the maintenance of extracellular Ca^2+^ can sustain Rem2 clustering after neuronal stimulation. Collectively our data indicate that Rem2 redistribution is mediated by Ca^2+^ influx through NMDARs.

### Molecular Determinants of Rem2 Redistribution are Localized to the C-terminus

**Figure 3:**
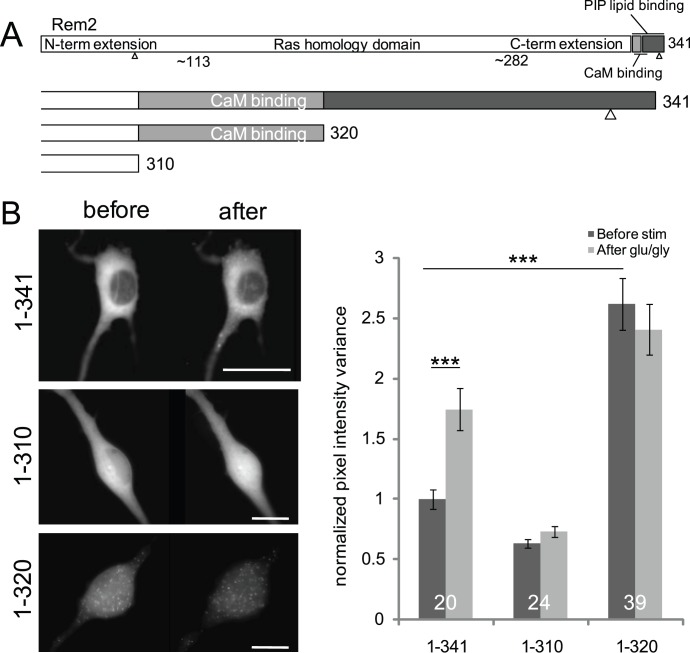
The Rem2 C-terminus directs redistribution. (**A**) Schematic of the Rem2 protein. The last 30 residues of the C-terminal extension contain a previously identified PIP lipid binding domain as well as a calmodulin binding site. Small triangles in the N-and C-termini represent 14-3-3 binding sites. We created truncated Rem2 proteins ending at residues 310 and 320 to determine if these interaction sites are relevant for Rem2 redistribution. (**B**) Effect of Rem2 truncations on redistribution. (**Left panel**) Full-length Rem2 (top) redistributed into puncta on glutamate/glycine stimulation. 1–310 Rem2 (center) showed no redistribution upon stimulation, while 1–320 Rem2 (bottom) formed puncta constitutively. Scale bars indicate 5 µm. (**Right panel**) WT Rem2 shows a significant difference in pixel intensity variance after stimulation (normalized mean (± SEM) pixel value variance before stimulation, 1.00±0,08; after, 1.74±0.17; paired t-test p<0.001). Additionally, 1–320 Rem2 distribution is different from WT before but not after stimulation (one-way ANOVA followed by Bonferroni’s test; before, p<0.001; after, p = 0.19). Pixel intensity variances were normalized to the variance of full-length Rem2 before stimulation.

The appearance of Rem2 puncta following NMDAR activation suggests that Rem2 may form interactions with Ca^2+^-binding proteins and/or undergo Ca^2+^-dependent self-association. The C-terminus of Rem2 contains several characterized molecular interaction domains, including a CaM-binding region, a polybasic domain that has been shown to bind PIP-lipids [16] and a 14-3-3 binding site. To examine the roles of the C-terminus and its ligands in redistribution, we created a series of C-terminally truncated CFP-tagged Rem2 proteins. We introduced stop codons at residues K311 and V321 to create Rem2 (1–310) and Rem2 (1–320), which lack the last 31 and 21 residues respectively (Figure 3A), and tested these mutants in a redistribution assay. Deleting the C-terminal residues at K311 (Rem2 1–310) completely abolished Rem2 redistribution. In contrast, Rem2 (1–320) formed puncta that were constitutive and not stimulation-dependent in neurons (Figure 3B) and were also present in transfected HEK cells (data not shown). A third mutant, Rem2 (1–330), behaved like full-length Rem2 in the redistribution assay, showing that the last 11 residues of Rem2, including the C7 motif, are not involved in the clustering of the protein (data not shown). Altogether, these data indicate that residues 320–330 may contain an auto-inhibitory domain that normally prevents the translocation of Rem2 into puncta, and that residues 310–320 contain a domain that is essential for the formation of Rem2 clusters inside the cell.

### Calmodulin Binding is Essential for Rem2 Redistribution

**Figure 4:**
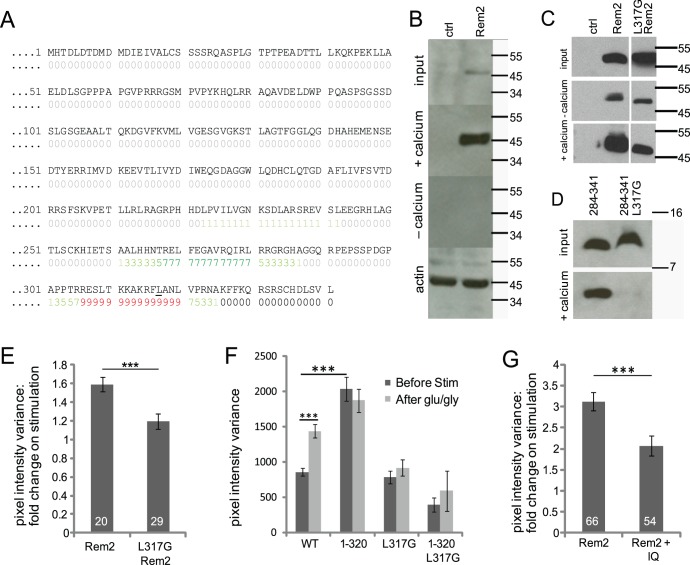
Calmodulin binding is necessary for Rem2 redistribution. (**A**) Predicted calmodulin binding sites in Rem2. An online predictor of calmodulin binding sites (http://calcium.uhnres.utoronto.ca/) suggests that Rem2 has two predominant sites, a higher affinity one close to the C-terminus and a lower affinity one further upstream. The colored numbers under the residues indicate how strongly a stretch of residues is predicted to bind CaM: grey 0 indicates no binding predicted, while red 9 indicates a likely CaM binding site. (**B**) Rem2 interacts with CaM in the presence of calcium. Untransfected HEK cells (ctrl) and HEK cells expressing full-length HA-tagged Rem2 (Rem2) were lysed, the lysates (**input**) cleared and passed over calmodulin-conjugated agarose beads in the presence of 3 mM calcium (**+calcium**) or 2 mM EGTA/2 mM EDTA (**–calcium**). Beta-actin was used as a loading control (**actin**). The eluate was subjected to Western blot analysis using HA antibody (top three panels) or actin antibody (bottom panel). (**C**) The point mutation L317G does not affect the association of full-length Rem2 with CaM. HEK cell lysates containing wild-type HA-Rem2 or the point mutation L317G were used for a pulldown assay with CaM-sepharose beads. Precleared lysates (**input**) and the eluates pulled down in buffer containing 2 mM EDTA/2 mM EGTA in place of calcium (**−calcium**) or 3 mM CaCl_2_ (**+calcium**) were separated by SDS-PAGE and transferred to nitrocellulose membranes, then probed with anti-HA antibody. Control cells are untransfected. Note less signal in the presence of EDTA/EGTA. (**D**) The same mutation abolishes interaction between CaM and the Rem2 C-terminal extension. Upper panel shows cleared lysates (input) from HEK cells expressing Rem2 (284–341) and Rem2 (284–341) L317G. Lower panel (+ calcium) shows Rem2 protein pulled down with CaM-sepharose in the presence of 2 mM calcium. (**E**) Rem2-calmodulin interaction is necessary for Rem2 redistribution. Neurons expressing either wild-type or Rem2 (L317G) protein were imaged before and after stimulation with 100 µM glutamate/10 µM glycine. Ratio after/before stimulation of WT Rem2, 1.59±0.07, N = 20; Rem2 (L317G), 1.19±0.08, N = 29; t-test p = 0.001. (**F**) Pixel intensity variances are shown before (dark bars) and after (light bars) stimulation. The variance of full length Rem2 changes upon stimulation (before, 858±53; after, 1429 96, N = 29; paired t-test p<0.001). The truncated mutant Rem2 (1–320) shows high variance before and after stimulation (before, 2035±165; after, 1871±161, N = 39), indicating constitutive puncta, while the Rem2 L317G and the 1–320 truncation carrying the L317G mutation show low variances (Rem2 L317G before, 784±87; after, 918±117, N = 29; Rem2 (1–320) L317G before, 392±101; after, 590±283, N = 10), indicating no constitutive puncta and little redistribution on stimulation. One-way ANOVA + Bonferroni post-hoc test comparing variances before stimulation shows significant differences between full length Rem2 and Rem2 (1–320) (p<0.0001), and no difference between full length and Rem2 L317G or Rem2 (1–320) L317G (p = 1 and 0.42, respectively). Rem2 (1–320) L317G data come from 10 cells from a single transfection. (**G**) Coexpression of a CaM-binding IQ motif reduces Rem2 redistribution. Neurons cotransfected with YFP-Rem2 and CFP-IQ (TVGKFYATFLIQEYFRKFKKRKEQ) were imaged before and after stimulation with 25 µM glutamate/2.5 µM glycine. Ratio after/before stimulation of Rem2 alone, 3.12±0.22, N = 66; Rem2+ IQ motif, 2.07±0.23, N = 54; t-test p = 0.0013.

Given the Ca^2+^ dependence of Rem2 redistribution and that Rem2 is capable of binding CaM (Moyers et al, 1997), we hypothesized that the Ca^2+^ dependence of Rem2 trafficking may be mediated by CaM. A CaM-binding site predictor highlighted two likely CaM-binding regions in Rem2, a higher-affinity site around residues 310–320, and a lower-affinity site around residues 280–290 (Figure 4A). Consistent with previous findings, HA-tagged Rem2 binds CaM in a Ca^2+^-dependent manner (Figure 4B). It was previously shown that a single point mutation in Rem2 (L317G) destabilizes the interactions between Rem2 and CaM [7]. Full-length Rem2 with the L317G mutation bound CaM robustly, possibly due to the predicted lower-affinity site (Figure 4C) or another site that was not predicted by the algorithm. In contrast, CaM-binding to a C-terminal fragment of Rem2 (Rem2 (284–341)) was abolished when the leucine residue was mutated (Figure 4D), indicating that the region around L317 indeed forms a CaM-binding site in Rem2. In spite of the robust biochemical interaction between full length Rem2 (L317G) and CaM, this mutant showed significantly reduced stimulation-induced clustering when expressed in neurons (Figure 4E), suggesting that an interaction between Rem2 and CaM near residue 317 is necessary for Rem2 redistribution. Finally, Rem2 (1–320) L317G did not form constitutive puncta and did not undergo redistribution after stimulation (Figure 4F), again supporting an important role for CaM in the trafficking of Rem2.

Our data thus indicate that the redistribution of Rem2 may be linked to its interaction with CaM. To obtain further evidence for the putative importance of this interaction, we created a small CFP-tagged peptide based on the IQ motif of the voltage gated Ca^2+^ channel Cav1.2. This IQ motif is a CaM-binding stretch of 24 residues and interacts with either or both the N-and C-lobes of Ca^2+^-CaM [17]. We reasoned that the overexpression of this CFP-IQ motif might competitively inhibit CaM interactions with Rem2. Indeed, when YFP-Rem2 was co-expressed with this motif, redistribution in response to stimulation with 25 µM glutamate/2.5 µM glycine was attenuated (Figure 4G). The results suggest that following NMDAR-dependent Ca^2+^ influx, Ca^2+^-CaM binds the region of Rem2 around residue L317, leading to its redistribution.

### Rem2 Interacts and Colocalizes with CaMKII

CaMKII redistributes rapidly in response to glutamate/glycine stimulation. This change in localization is dependent on NMDAR activation and Ca^2+^/CaM activation [2], [18], [19]. Because Rem2 redistributes under identical conditions, we hypothesized that the two phenomena might be linked. This hypothesis was tested by both biochemical and imaging means.

**Figure 5:**
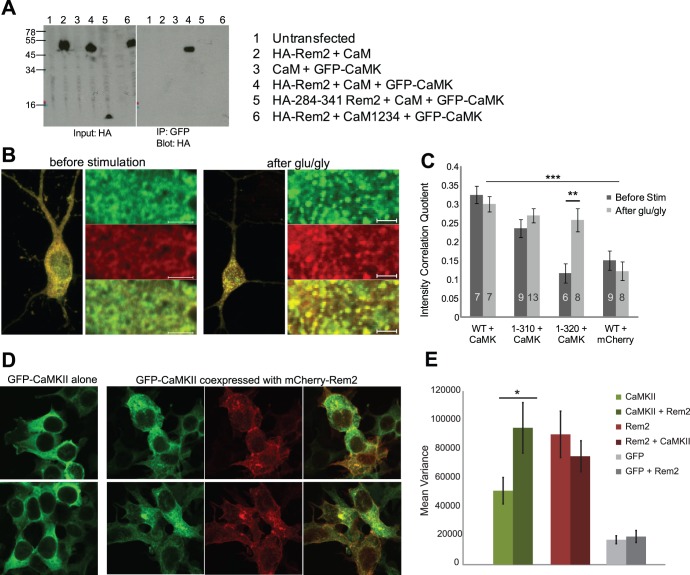
Rem2 interacts and redistributes with CaMKII. (**A**) Calcium-calmodulin is essential for Rem2-CaMKII association. HEK cells were transfected with the indicated GFP-CaMKII, CaM, the calcium-insensitive mutant CaM1234 or HA-Rem2 constructs. Cell lysates were coprecipitated with anti-GFP antibody coupled to proteinA/G beads. Coprecipitated proteins were eluted from beads in sample buffer and separated using 10%/16% tricine SDS-PAGE followed by Western blotting with anti-HA antibody. Precleared lysates (**left**) and coprecipitated proteins (**right**) are shown. CaMKII binds only to full length Rem2 (lane 4) and not to a C-terminal fragment of Rem2 (lane 5). CaMKII also shows no binding when Rem2 and CaMKII are coexpressed with CaM1234 (lane 6). (**B**) Fluorescent signals of GFP-Rem2 and mCherry-CaMKII redistribute together. Rem2 and CaMKII cotransfected neurons were fixed following no stimulation or 60 seconds of 100 µM glutamate/10 µM glycine stimulation and 5 minutes of recovery. Left subpanel of each panel shows a cell coexpressing CaMKII and Rem2. Right subpanel shows a higher resolution of the subcellular distribution of GFP-Rem2 (top), mCherry-CaMKII (center) and an overlay (bottom). Scale bars are 5 µm. (**C**) Rem2 and CaMKII are colocalized before (dark bars) and after (light bars) stimulation. Intensity correlation of GFP and mCherry signal is given for neurons coexpressing mCherry-CaMKII and GFP-Rem2 wild-type or C-terminal truncations. The higher the intensity correlation quotient (ICQ), the greater the covariance of the two colors across the cell, i.e. higher colocalization within the cell. Correlation of GFP-WT Rem2 and unconjugated mCherry is given as control. ICQ of GFP-WT-Rem2+ mCherry-CaMKII before stimulation, 0.312±0.015; GFP-WT-Rem2+ mCherry alone, 0.137±0.018; two-way ANOVA, p<0.001. Only the constitutively punctate 1–320 Rem2 mutant shows a different distribution from CaMKII before stimulation, although it correlates with CaMKII after. Before stimulation, 0.117±0.026; after stimulation, 0.258±0.030; t-test, p = 0.004. (**D**) Rem2 alters basal CaMKII distribution. HEK cells expressing GFP-CaMKII alone (left panels) or GFP-CaMKII coexpressed with mCherry-Rem2 (right panels) were fixed and imaged using confocal microscopy. Note the diffuse distribution of GFP-CaMKII when Rem2 is absent. (**E**) Rem2 alters CaMKII distribution. Mean pixel intensity variance in fixed HEK cells of GFP-CaMKII (alone, 51106±9158, N = 69; with Rem2, 94591±17452, N = 56), mCherry-Rem2 (alone, 90085±16053, N = 60; with CaMKII 74948±10689, N = 60) or GFP fluorescence (alone, 17232±2775, N = 60; with Rem2, 19442±4145, N = 57).

When co-expressed with CaM in HEK cells, GFP-CaMKII coprecipitated with HA-Rem2 in the presence of Ca^2+^. Experiments involving C-terminal deletions of Rem2 revealed that CaMKII also interacted with a Rem2 fragment encompassing residues 1–149 (data not shown), indicating that CaMKII interacts with Rem2 at a site distinct from the CaM interaction region. Indeed, CaMKII did not bind to the C-terminal fragment Rem2 (284–341) (Figure 5A, lane 5). Interactions between WT Rem2 and CaMKII were less robust when CaM was omitted, and did not occur at all when CaM was replaced with CaM1234, a Ca^2+^-binding deficient mutant, suggesting that Ca^2+^-CaM is an essential component of this complex (Figure 5A, compare lanes 4 and 6). Because the interaction between Rem2 and CaMKII occurred in the absence of the main CaM interaction site on Rem2, the dependence of the interaction on CaM may be due to CaM binding to CaMKII rather than to Rem2, although we cannot exclude the possibility that Rem2 has an additional CaM interaction site towards the N-terminus. We also attempted to co-immunoprecipitate native Rem2 and CamKII from whole brain tissue, however, the unavailability of high quality Rem2 antibodies precluded this experiment.

We next performed live imaging on rat hippocampal neurons co-transfected with GFP-Rem2 and mCherry-CaMKII. Figure 5B shows extensive colocalization between Rem2 and CaMKII both before and after stimulation. Subcellular distribution of Rem2 showed greater covariance with mCherry-CaMKII than with mCherry alone. Rem2 (1–320) also colocalized with CaMKII, but only after stimulation, showing that upon stimulation CaMKII may be drawn to the constitutive puncta formed by Rem2 (1–320) (Figure 5C).

Because neurons contain large amounts of endogenous CaMKII [20], [21] and significant amounts of Rem2 [12], we could not determine whether the distribution of the transfected fluorescent proteins was affected by the presence of endogenous proteins. To simplify the experimental environment, we expressed GFP-CaMKII and mCherry-Rem2 in HEK cells and examined the subcellular localization of CaMKII in the presence and absence of co-expressed Rem2. GFP-CaMKII showed diffuse fluorescence that was similar to GFP alone, except for the exclusion from the nucleus. When mCherry-Rem2 was cotransfected, however, the distribution of GFP-CaMKII signal became more heterogeneous, and strongly overlapped with mCherry-Rem2 (Figure 5D), suggesting that Rem2 can direct CaMKII to specific subcellular compartments or components, where it resides. By contrast, the distribution of mCherry-Rem2 fluorescence in the cells did not change when CaMKII was co-expressed (Figure 5E). Thus, Rem2 can alter the distribution of CaMKII under basal intracellular Ca^2+^ levels but CaMKII does not affect Rem2 distribution.

**Figure 6:**
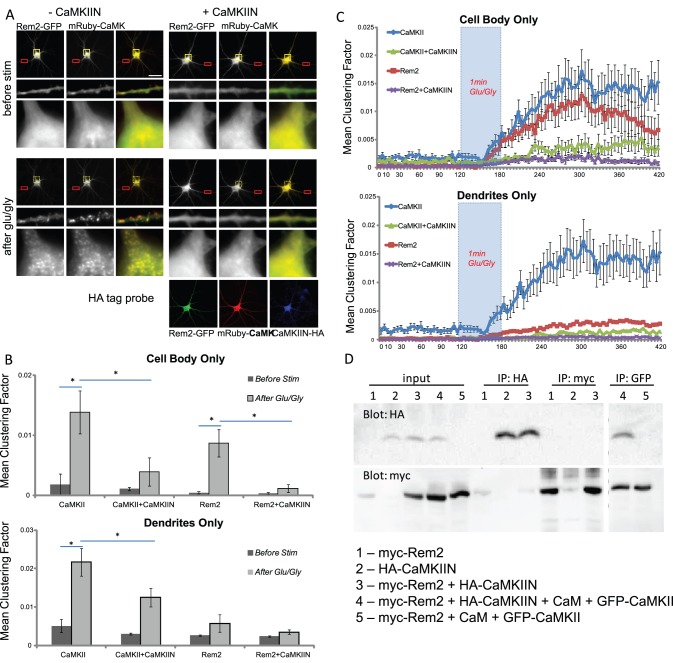
Rem2 and CaMKII redistribution is co-dependent. (**A**) CaMKIIN inhibits redistribution of Rem2 and CaMKII. Rat hippocampal neurons coexpressing **(**left) GFP-Rem2 and (right) mRuby-CaMKII without (left panels) and with (right panels) HA-CaMKIIN. Cells were imaged before stimulation (top panels), then stimulated with 100 µM glutamate/10 µM glycine and imaged again (middle panels). Neurons were fixed and probed for HA to detect CaMKIIN (bottom panel). The scale bar represents 20 µm. (**B**) CaMKIIN reduces redistribution of coexpressed CaMKII and Rem2. Clustering factor determined as in [2]. Mean ± SEM clustering factor for CaMKII without CaMKIIN: before stimulation, 0.004±0.002; after, 0.021±0.004; with CaMKIIN: before, 0.002±0.0002; after, 0.009±0.002. Rem2 clustering without CaMKIIN: before stimulation, 0.001±0.0002; after, 0.009±0.002; with CaMKIIN: before, 0.001±0.0002; after, 0.003±0.0006. Data is from 4 separate experiments with a total of N = 27 neurons/condition. Error bars are ± SEM; asterisks represent p<0.05 (Kruskal-Wallis test followed by Tukey’s post hoc test). (**C**) Rem2 and CaMKII redistributions overlap temporally. A timecourse of aggregation of Rem2 and CaMKII shows little clustering before stimulation with glutamate/glycine, and Rem2 and CaMKII clustering occurs at similar rates. N = 27 neurons/condition. (**D**) CaMKIIN does not interact with Rem2, and expression of CaMKIIN does not interfere with Rem2-CaMKII interaction. HEK cells were transfected with the indicated plasmids. Cell lysates were subjected to co-precipitation assays using a mix of proteinA/G beads and anti-HA,-myc or-GFP. Eluates were separated on SDS-PAGE and probed with anti-HA (top) and anti-myc (bottom). (**E**) CaMKII aggregates in HEK cells following a pH drop/high Ca protocol [2] (mean clustering factor before simulation 0.030±0.002; after 0.166±0.013). Aggregation is inhibited by CaMKIIN (before stimulation, 0.024±0.002; after, 0.068±0.010) but unaffected by coexpression of Rem2 (before, 0.031±0.003; after, 0.152±0.009). CaMKIIN inhibition is also insensitive to Rem2 coexpression (before, 0.031±0.003; after, 0.071±0.006). N = 20 cells and p<0.05 for all before-after pairs. (**F**) Rem2 does not aggregate using the same protocol (without CaMKIIN: before stim, 0.037±0.004; after, 0.032±0.004; with CaMKIIN: before, 0.018±0.002; after, 0.022±0.003), unless CaMKII is also present (without CaMKIIN: before stim, 0.012±0.002; after, 0.073±0.008). CaMKII-induced Rem2 aggregation is also inhibited by CaMKIIN (before, 0.008±0.001; after, 0.021±0.004). Error bars are ± SEM; asterisks represent p<0.05 (Kruskal-Wallis test followed by Tukey’s post hoc test).

To determine if there is a reciprocal CaMKII effect on Rem2 trafficking, we used an inhibitor of CaMKII aggregation to examine its consequences on Rem2 distribution. CaMKII aggregation is thought to occur when the autoinhibitory domain of a CaMKII subunit of one holoenzyme binds to the catalytic domain of a subunit of an adjacent holoenzyme [2], [22]. CaMKII activity and aggregation are inhibited by CaMKIIN, a 79-amino acid natural peptide that binds specifically to the catalytic pocket of activated CaMKII [23], [24]. We co-expressed mRuby-CaMKII and GFP-Rem2 in neurons with and without CaMKIIN and imaged cells before and after stimulation. Co-expression of CaMKIIN substantially reduced aggregation of both CaMKII and Rem2 (Figure 6A & B), suggesting that CaMKII contributes to Rem2 redistribution. Moreover, the time course of puncta formation following glutamate/glycine stimulation was similar for mRuby-CaMKII and GFP-Rem2 (see Movie S1), both in the absence and presence of the inhibitor (Figure 6C). CaMKIIN effectively reduced CaMKII clustering in both the cell body and dendrites. Rem2 clustering was much more prominent in the cell bodies, whereas it occurred variably in spines, and CaMKIIN also inhibited Rem2 aggregation (Figure S2).

Because there is some sequence similarity between the C-terminus of Rem2 and the auto-inhibitory region of CaMKII, we wanted to ensure that the effect of CaMKIIN on Rem2 aggregation was mediated through CaMKII rather than Rem2. To do this, we performed a co-immunoprecipitation assay with Rem2 and CaMKIIN in the presence and absence of Ca^2+^-CaM/CaMKII. HA-CaMKIIN did not interact with myc-Rem2, and did not interfere with the interaction between myc-Rem2 and GFP-CaMKII (Figure 6D), showing that the effects of CaMKIIN were mediated via CaMKII and not as a result of a direct inhibitory effect on Rem2.

We further examined the relationship between Rem2, CaMKII and CaMKIIN in HEK cells using a stimulation protocol that previously allowed dissecting the mechanisms of CaMKII clustering [2]. Following this protocol, GFP-CaMKII expressed alone formed puncta, whereas mCherry-Rem2 expressed alone did not. However, co-expression of CaMKII and Rem2 resulted in co-aggregation. As expected, co-expression of CaMKIIN inhibited clustering of CaMKII as well as co-clustering of CaMKII and Rem2 (Figure 6E and F, Figure S3). Thus, Rem2 alone does not aggregate in HEK cells, but it forms puncta when CaMKII is present, and this co-aggregation is inhibited by CaMKIIN. These data indicate that CaMKII is an important determinant of Rem2 clustering.

## Discussion

The small GTPase Rem2 is one of several members of the family of RGK proteins that also includes Rem, Rad, and Gem/Kir. Investigations concerning the functional roles of these proteins have focused mainly on their regulation of high voltage activated Ca^2+^ channels in heart, muscle and brain ([25]–[28]; for review, see [29]). However, relatively little is known about other physiological functions of this family of proteins. Here, we show that Rem2 undergoes activity-dependent changes in subcellular distribution, and that this effect is mediated by NMDAR-dependent activation of CaM (Figures 1 and 2). Furthermore, we show that Rem2 colocalizes and co-traffics with CaMKII, thus hinting at a possible role of Rem2 in the well documented effects of CaMKII on neuronal plasticity (reviewed in [30], [31]).

**Figure 7:**
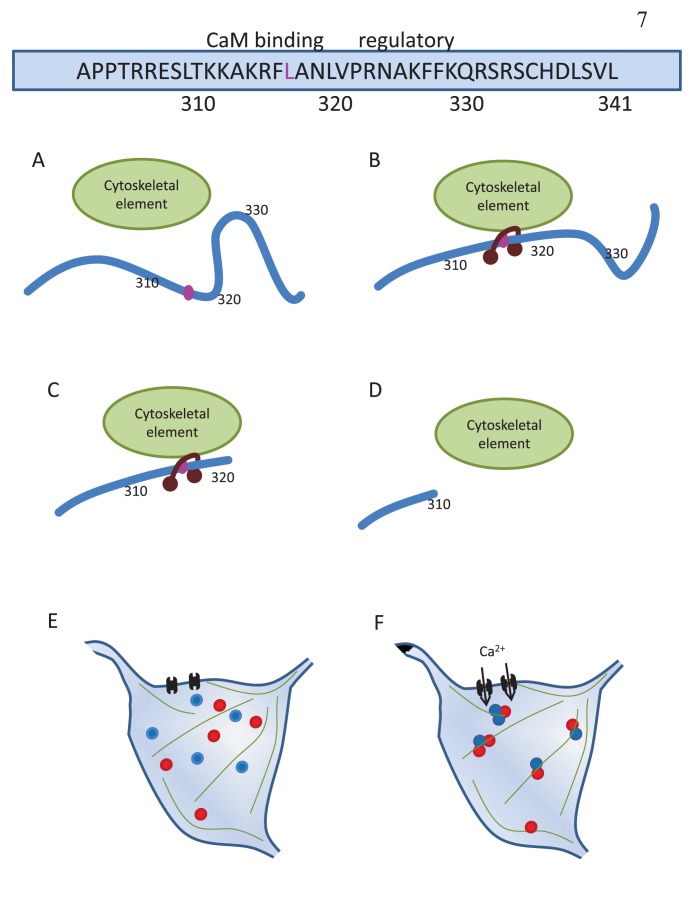
Possible mechanism underlying Rem2 activity-dependent redistribution. Top: Representation of the C-terminus of Rem2, with the putative CaM-binding determinant leucine residue highlighted in pink. (A) In the absence of stimulation, Rem2 residues 320–330 (regulatory region) may allosterically inhibit Rem2 association with a putative cytoskeletal regulatory element, and consequently no Rem2 puncta are observed. (B) Upon stimulation, Ca^2+^-CaM may bind the region around residue L317, allowing redistribution to occur by association with the cytoskeleton. (C) The Rem2 mutant 1–320 lacks the regulatory region. This would result in constitutive association with CaM and constitutive puncta. (D) The Rem2 mutant 1–310 lacks the CaM-binding region, and would not redistribute to puncta unless CaMKII is overexpressed. (E) Under basal conditions, Rem2 (blue circles) and CaMKII (red circles) are diffusely distributed in neurons. (F) Upon neuronal stimulation, Ca^2+^ influx through the NMDAR (black brackets) leads to aggregation of Rem2 and CaMKII, possibly at cytoskeletal elements (green lines).

We identified two regions of the Rem2 C-terminus that appear to be involved in the regulation of redistribution (Figure 3). The truncated Rem2 mutant 1–310 was incapable of undergoing redistribution upon stimulation, while the 1–320 mutant showed constitutive puncta that did not change substantially upon stimulation. These data imply that the residues in the 311–320 region comprise a domain that is essential for stimulation-induced redistribution. A critical residue in this sequence, L317, appears to be necessary both for CaM binding and for redistribution, suggesting that CaM interactions with Rem2 at this site are a prerequisite for Rem2 trafficking (Figure 4). This fits with our observation that the redistribution of Rem2 required Ca^2+^ and that Rem2 interacted biochemically with Ca^2+^-CaM, but not apoCaM. The adjacent region spanning residues 321–330 appears to form a regulatory domain to mediate auto-inhibition of Rem2 trafficking under basal (i.e., non-stimulated) conditions, as removal of this region resulted in constitutive Rem2 puncta. We thus envision a mechanism in which Ca^2+^-CaM interactions with residues 310–320 result in a conformational change in Rem2 that in turn removes the auto-inhibition mediated by residues 320–330 (see Figure 7). This is reminiscent of what has been described for CaMKII, in which an autoregulatory region is adjacent to the CaM-binding domain (reviewed in [22]).

The cellular determinants known to be involved in CaMKII translocation in neurons also appear to be required for Rem2 translocation. This includes a reliance on NMDAR-mediated Ca^2+^ entry and dependence on Ca^2+^-bound CaM. Full-length Rem2 also immunoprecipitated with CaMKII, as did substantially C-terminally truncated Rem2 proteins lacking the main CaM interaction site, consistent with similar experiments using the related RGK protein Rad [10]. Following activation of NMDARs, Rem2 translocated to the same subcellular loci as CaMKII (Figure 5B and C), suggesting that Rem2 and CaMKII traffic as a protein complex.

This then begs the question as to whether the trafficking of Rem2 and CaMKII are influenced by one another. The cellular distribution of these proteins correlated in neurons and in HEK cells, a first suggestion that one of the two proteins might impact the localization of the other. To determine if this is the case, we simply examined whether expression of one influenced the cellular distribution of the other (and vice versa) in HEK cells. Rem2 did affect the baseline distribution of CaMKII, but not the reverse (Figure 5D and E). Thus at basal Ca^2+^ levels, Rem2 might be restricting CaMKII to specific subcellular regions. This analysis could not be done in neurons because of pre-existing levels of both native proteins, but if this conclusion also applies to neurons, Rem2 could directly influence the nature of Ca^2+^ signals that CaMKII is exposed to. Indeed, the source of Ca^2+^ entry into the cytosol is a critical determinant in the downstream signaling mechanisms that are activated by the second messenger. One way to mediate this spatial specificity of Ca^2+^ signaling is to place and hold the signaling machineries at specific sites, a role that Rem2 might fill for CaMKII signaling. Given that RGK proteins can alter cell morphology by regulating the actin and microtubule cytoskeletons [8], [32]–[38], and Rem2 overexpression leads to neurite outgrowth [7], it is possible that Rem2 helps anchor CaMKII to cytoskeletal elements, thus potentially facilitating CaMKII-mediated insertion of NMDARs. We propose that Rem2 may help retain a significant fraction of CaMKII in subcellular domains in neurons under basal conditions. Following NMDAR activation, CaMKII and Rem2 move together into clusters, likely as part of a larger protein complex.

During NMDAR activity, CaMKII can translocate to synaptic and extra-synaptic sites, and it was proposed that the multivalent nature of CaMKII could support co-aggregation with additional binding partners in large complexes [2]. Indeed, we found that stimulation of HEK cells to induce CaMKII clustering also caused co-clustering of Rem2 (Figure S3). Additionally, in neurons, we found that 1–310 Rem2 could form clusters in the presence of overexpressed CaMKII but not its absence (Figure 5C). Furthermore, co-expression of the natural inhibitor of CaMKII, CaMKIIN, reduced the co-clustering of both proteins in HEK cells and neurons (Figure 6, Figure S3). Thus, CaMKII seems to be an important determinant of Rem2 redistribution. On the other hand, co-expression of Rem2 altered the subcellular distribution of CaMKII in HEK cells, indicating that Rem2 can potentially direct CaMKII to specific cellular compartments. Furthermore, upon neuronal stimulation, CaMKII forms clusters at the constitutive puncta of 1–320 Rem2, further suggesting that aggregation of Rem2 can attract CaMKII to focal points. The sites to which Rem2 might attract CaMKII inside neurons have not been precisely determined. Ghiretti and Paradis (2011) performed immunohistochemistry with a custom-designed antibody and showed that Rem2 was expressed throughout somato-dendritic domains, yet did not seem particularly enriched in spines. This fits with our observation that the majority of Rem2 clusters were found in the cell body rather than in the dendritic compartment.

Knockdown of Rem2 leads to reduced numbers of synapses in developing neurons as well as alterations in dendritic and synaptic development, spine shape and stability [12], [39]. Because many of these functions have been also attributed to CaMKII [14], [40]–[42], we can speculate that the co-trafficking of Rem2 and CaMKII may support some of these processes. For example, the NMDAR-dependent translocation of CaMKII is thought to have important roles in synaptic plasticity and remodelling. In this context, our evidence that Rem2 co-aggregates with CaMKII may indicate a collaborative role between CaMKII and Rem2 in synaptic development and plasticity. Such a role for Rem2 in regulating neuronal function may well involve voltage-gated Ca^2+^ channels, a major target of RGK protein signaling (reviewed in [3]).

### Conclusions

We have shown here that the small GTPase Rem2, expressed ectopically in neurons, redistributes upon neuronal stimulation under conditions almost identical to those which are required for CaMKII redistribution. We also found a biochemical interaction between Rem2 and CaMKII in the presence of Ca^2+^-CaM. Rem2 and CaMKII co-distribute in neurons before and after stimulation, and an inhibitor of CaMKII self-association also inhibits Rem2 redistribution. This co-trafficking of Rem2 and CaMKII suggests that Rem2 may modulate the subcellular positioning of CaMKII, thus potentially affecting its function within neurons.

## Materials and Methods

### DNA Constructs

Rem2 cDNA was amplified by RT-PCR from rat brain mRNA and cloned into the pCMV-HA (Clontech) expression vector, creating N-terminally HA-tagged Rem2. C-terminal Rem2 truncations were made using site-directed mutagenesis to introduce premature stop codons. N-terminally truncated Rem2 constructs were generated by PCR followed by cloning into the pCMV-HA vector. All Rem2 constructs to be used for imaging were subcloned from pCMV-HA into pECFP, pEYFP or pEGFP (Clontech). mCherry-Rem2 was created by cloning an mCherry cassette in place of CFP in CFP-Rem2. mCherry-CaMKII and mRuby-CaMKII were made by cloning mCherry or mRuby into mGFP-CaMKII [2]. CaMKIIN was amplified from a mouse hippocampal cDNA library (RIKEN) and cloned into mGFP to make GFP-CaMKIIN. This fusion product was then moved into pcDNA3 behind the CMV promoter and the GFP was replaced with an HA coding sequence made from annealed oligonucleotides. The CFP-IQ motif was made by cloning annealed oligos into pECFP. We received the following expression vectors as kind gifts: CaM and CaM1234 from Dr. John Adelman (Oregon Health & Science University) and mRuby from Dr. Jörg Wiedenmann (Institute of General Zoology and Endocrinology, University of Ulm, Germany).

### Cell Culture and Transfection

tsA-201 HEK293 cells were acquired from ATCC and maintained as recommended (37C, 5% CO_2_, DMEM supplemented with 10% FBS, 2 mM L-glutamine and 100 U/ml penicillin/100 µg/ml streptomycin). For biochemistry experiments, we transfected cells at 30–50% confluence using calcium phosphate, then washed them the next morning and refreshed the media. For Rem2 and CaMKII fluorescence imaging experiments, we transfected at 30% confluence using Lipofectamine 2000 with some or all of the following plasmids: mGFP-CaMKIIα, Rem2-mCherry, HA-tagged CaMKIIN.

Primary hippocampal neurons were taken from P0 Sprague-Dawley rat pups (Charles River) and cultured essentially as described in [43] or Hudmon et al, 2005. Briefly, hippocampi were digested with papain then triturated in neuronal media to dissociate them. Cells were plated on either silicon wafers for the redistribution assay or on poly-D-lysine-coated coverslips for confocal microscopy. Neurons were transfected using Lipofectamine 2000 and 2 µg DNA per well of a 24-well plate.

### Co-immunoprecipitation, Pulldown Assays and Immunoblotting

Cells were harvested 48–72 hours after transfection and lysed using a gentle lysis buffer (10 mM Tris-HCl, 140 mM NaCl, 0.5% NP-40) with Complete EDTA-free protease inhibitor (Roche) for 20 minutes. For co-immunoprecipitations with no Ca^2+^, the lysis buffer contained 2 mM EDTA/2 mM EGTA; for co-immunoprecipitations with Ca^2+^, the lysis buffer contained 2 mM CaCl_2_. Cell lysates were precipitated with either a mix of protein A and protein G beads incubated with anti-GFP (Santa Cruz) or Sepharose beads covalently linked to calmodulin (GE Healthcare). We took “input” samples of cleared cell lysates before we added beads. Lysates were incubated with pre-washed beads and rotated overnight at 4C, then washed three times in lysis buffer. Bound proteins were eluted with 4X Laemmli buffer and separated on SDS-PAGE gels, then transferred to nitrocellulose membranes and subjected to Western blot analysis using anti-HA antibody (Roche).

### Confocal Microscopy

Fixed neurons on coverslips were mounted on a standard microscope slide using Cytoseal XYL (Apogent). Confocal images were collected on a Zeiss LSM-510 Meta inverted microscope using a 63x 1.4NA oil immersion objective. Z-stacks were taken through the thickness of cells. To image YFP, GFP and CFP we excited with an argon laser at 514, 488 and 458 nm, respectively, and for mCherry and Alexa-594 we excited with a helium-neon laser at 543 nm. To visualize GFP and mCherry, we used long-pass filters at 505 and 560 nm, respectively.

### Redistribution Assay Using Epifluorescence Microscopy

Cells on silicon chips were transfected between days 10–14 with the desired DNA constructs, then tested 24 hours after transfection. To assay Rem2 redistribution, a chip was placed in a stimulation dish filled with warm EBS (135 mM NaCl, 3 mM CaCl_2_, 5 mM KCl, 2 mM MgCl_2_, 10 mM D-glucose, 10 mM HEPES, pH 7.3, osmolarity = 305–315 mOsm). This dish contained a square cutout that held the chip in a given orientation to facilitate marking cells’ positions. We then mounted the loaded dish on the stage of an Olympus BX61 upright fluorescent microscope, and imaged cells using a 100X 1.0 NA water immersion objective. Images were captured using a Watec N102 CCD camera with Astrovid software.

To examine Rem2 redistribution using bath application of stimulant, we first imaged and marked the positions of ∼10 cells per chip using a programmable X–Y mover (Sutter Instruments). We then added a stimulant solution, usually glutamate/glycine, for 60 seconds, then removed the stimulating solution and replaced it with EBS to allow the cells to recover. We re-imaged the marked cells starting 60–90 sec after recovery. These stimulation conditions were similar to those used to study CaMKII translocation in hippocampal neurons [2].

Photoconductive stimulation of Rem2-expressing cells was performed according to the protocol described in [15]. To stimulate individual cells, we imaged the cell bodies using a CFP filter, then stimulated the chip using 3–5 V of current for 2 msec at 20 Hz for 5 sec. Cells were imaged before and after stimulation to compare Rem2 distribution. When two groups were compared, for example, CFP-Rem2 vs CFP alone, cells in each group came from the same culture round (i.e. from the same dissection) to minimize variability within experiments. To ensure that differences between groups were robust and not simply due to an anomalous transfection or culture, in most cases groups were averaged over at least three transfections of at least two different culture rounds, unless otherwise stated.

### Propidium Iodide Staining for Dying Cells Following Stimulation

To determine if glutamate/glycine stimulation of Rem2 expressing cells caused rapid cell death, we stained cells with the DNA intercalating dye propidium iodide (PI). Immediately following a 60-second application of glutamate/glycine, the stimulating solution was replaced with EBS containing PI at 2 µg/ml. The chip or coverslip was incubated in this solution for 4 minutes prior to imaging. Images were taken at 100x before stimulation and again after stimulation and addition of PI.

### Analysis of Rem2 Redistribution with ImageJ convolution Filters

To minimize noise, our image-capture software averaged 8 images to get one “before stimulation” and one “after stimulation” image. All subsequent manipulations were performed in ImageJ (NIH) using default or specialized plugins. 8-bit images were compiled into an avi file and subjected to the Stackreg plugin followed by the Smooth function. In epifluorescence, the signal from diffuse Rem2 across the neuronal cell body was not consistent. Some parts of the cell were brighter than others, possibly reflecting the thickness of the cell at that point. Thus, although the Rem2 puncta that formed on stimulation appeared brighter than the background across the cell, subtracting the background from the signal posed a problem, because it was not uniform across the cell. To solve this, we used a simple spatial convolution filter which applied a 5×5 matrix to each pixel in the image. This filter multiplied the value of a given pixel (0–255) by 24, then subtracted the values of the 24 surrounding pixels and output the final value to a new image. If a given pixel was therefore about the same intensity as its neighbours, the filter would give a low output value. Conversely, if a pixel value were much higher than its neighbours of a 5×5 area, as is the case of a Rem2 punctum, the output value would be high. Thus, the convolution filter maximizes signal from small spots that are brighter than their surroundings, while minimizing fluctuations in background fluorescence due to cell thickness or other factors (Figure S4).

To quantify differences in Rem2 redistribution before and after stimulation, we determined the variance in the intensity values of the output images. Using ImageJ ROI tools, we drew non-overlapping regions of interest (ROIs) around as much of the cell interior as possible. Each ROI was measured to determine the pixel value mean and standard deviation, and area. Standard deviations were squared to get variances for each ROI, then ROI variances were averaged for a given cell. Cell averages were then used to create an average of the pixel value variance for a given condition. To determine the change in pixel value variance after stimulation, we calculated the ratio of the cell’s average variance after stimulation to before stimulation. These per-cell values were averaged for a given condition.

### Rem2–CaMKII Covariance Analyzed with ImageJ Intensity Correlation Analysis

To quantify Rem2-CaMKII colocalization, we used the Intensity Correlation Analysis (ICA) set of plugins bundled with ImageJ from the Wright Cell Imaging Facility at the University of Toronto. This protocol is detailed in [44], and determines an Intensity Correlation Quotient (ICQ) to quantify how much two fluorescent signals covary across a region of interest. The ICQ ranges from −0.5 (segregated signals) to 0 (random signals) to +0.5 (dependent signals). Confocal hyperstacks were converted to 8-bit, separated by color and then background-subtracted using a region of the image that did not contain a cell. ROIs were chosen with the caveat that the cell must fill the ROI in all slices of the stack. Slices taken from the top and very bottom of the cell were discarded for this reason. For fear of losing information, we did not threshold the images as recommended by the protocol. In this way we generated a single ICQ value for each cell, and averaged the cells in each condition.

### Rem2–CaMKII Redistribution in Neurons

To quantify the co-clustering of CaMKII and Rem2 in neurons we used primary rat hippocampal cultures plated at high density (1 million cells/coverslip) on 18 mm glass coverslips. We then cotransfected the cells at 12–14 DIV using Lipofectamine 2000 with mRuby-CaMKII and GFP-Rem2, with or without HA-CaMKIIN. The live imaging experiments were performed the following day on a Zeiss Axiovert inverted microscope using an oil 63x/1.4 NA objective and a Xenon lamp. Coverslips were washed for 2 min, stimulated with glutamate/glycine for 1 min and washed again for 4 min in a 37°C heated chamber with HBSS-based solution [2]. The number of clusters was measured on the time lapse images using a morphometric analysis using Meta-Morph software. A cluster was detected when a contiguous group of pixels with an area ranging from 0.1 to 1.0 µm^2^ was at least twice as bright as the average fluorescence of all the pixels in the cell and had a shape factor (4πA/P^2^; A, area; P, perimeter) >0.5. The clustering factor is defined as the total brightness of all clusters divided by the fluorescence of the whole cell.

### CaMKIIN Expression

To confirm the expression of the HA-tagged CaMKII inhibitor, cells were fixed immediately after the end of the live imaging in 37°C 4% paraformaldehyde solution (0.1 M PB, ph 7.4, 4% sucrose, 2 mM EGTA, pH 7.4) for 10 minutes and then washed twice in PBS and once in PBS/0.1 M glycine for 10 minutes. For immunostaining, the coverslips were incubated 30 min in blocking solution (PBS, 2% goat serum and 0.05% Triton X-100), 2 hours in rabbit anti-HA (clone DW2, 1∶500, Upstate) and 45 minutes in goat anti-rabbit ATTO 647 (1∶1000, Alexis Biochem), then mounted with Prolong Gold (Invitrogen). We imaged the coverslips on a Zeiss LSM510 META confocal microscope using an oil 63x/1.4 NA objective. GFP, mRuby and ATTO647 fluorophores were excited at 488 nm, 543 nm and 633 nm respectively.

### Rem2 and CaMKII Localization and Redistribution in HEK Cells

To determine whether Rem2 affects CaMKII distribution in HEK cells (or vice-versa), we fixed HEK cells that were transfected (24 hr before, using Lipofectamine 2000) with either one or a combination of the following plasmids: mGFP-CaMKII, mCherry-Rem2 and mGFP. Confocal images were analysed using Meta-Morph software to evaluate the standard deviation of the fluorescence distribution within ROIs taken from 20–30 HEK cells per condition. The mean variance for each condition was calculated as described above for Rem2 redistribution.

To assess the impact of Ca^2+^ stimulation on Rem2 and CaMKII clustering in HEK cells, we used a Ca^2+^/ionomycin + nigericin-based protocol, followed by cell fixation and confocal imaging, as described in [2].

### Statistical Analysis

All analyses were performed using Microsoft Excel, Origin Pro 8.0 or MatLab.

## Supporting Information

Figure S1 **Rem2 redistribution is independent of epitope tag.** (**A**) Confocal images of GFP-Rem2 (top) and HA-Rem2 (center) and their overlay (bottom) after stimulation of a neuronal culture. Neurons were stimulated for 60 seconds with 100 µM glutamate/10 µM glycine, recovered for 5 minutes then fixed in 4% paraformaldehyde. Cells were then probed with rat anti-HA antibody followed by anti-rat secondary conjugated to Alexa-594. (**B**) Intensity Correlation Analysis of the overlap between Alexa-594 and GFP signals in glutamate/glycine-stimulated cells transfected with HA-Rem2+ GFP or HA-Rem2+ GFP-Rem2. Puncta formed by GFP-Rem2 overlapped those formed by HA-Rem2 (ICQ for HA-Rem2+ GFP alone, 0.085±0.007, N = 5; HA-Rem2+ GFP-Rem2, 0.283±0.026, N = 8; t-test p = 0.0001), indicating that the Rem2 foci were consistently formed upon stimulation, regardless of the molecular tag.
(EPS)

Figure S2 **CaMKII and Rem2 clusters in dendrites and cell body.** Data represented in Figure 6C are broken down here by cellular compartment. The upper panels show the mean clustering factor of CaMKII and Rem2 in the presence and absence of CaMKIIN in dendrites, while the lower panels show clustering in the cell body. Note that Rem2 clustering in the dendrites persists, while clusters in the cell body begin to dissipate after about 5 minutes.
(EPS)

Figure S3 **CaMKII drives Rem2 aggregation in HEK cells.** (**A**) HEK cells transfected with Rem2, CaMKII or both show no aggregation under basal conditions (upper panels). In response to a pH drop/ionomycin protocol, CaMKII forms robust aggregates, while Rem2 does not (lower panels). Rem2 can aggregate upon stimulation in the presence of CaMKII. (**B**) CaMKIIIIN inhibits CaMKII aggregation upon stimulation (left panel set), while Rem2 is unchanged by CaMKIIIIN (right panel set). Coexpression of these three plasmids results in lowered CaMKII-Rem2 co-aggregation (bottom panel set). Scale bar represents 10 µm.
(EPS)

Figure S4 **Convolution filtering of acquired images.** (**A**) The convolution filter used here was a 5×5 matrix applied to each pixel of the image. The value of the pixel was multiplied by 24, then the surrounding 24 pixel values were subtracted. The resulting pixel value was output to a new image. Because this is an edge-detection algorithm, we avoided drawing ROIs that included an edge, either the cell membrane or the nuclear membrane. (**B**) Images taken before and after stimulation (upper left and upper right, respectively) show the background variation in pixel value. Bottom left and right images depict the upper images following application of the convolution filter. Note that variation in fluorescence background is removed while the signal from the puncta is left intact.
(EPS)

Movie S1 **Rem2 and CaMKII redistribute together.** Cultured hippocampal neurons cotransfected with GFP-Rem2 and mRuby-CaMKII were imaged using epifluorescence every 5 seconds for 7 minutes. Stimulation with 100 µM glutamate/10 µM glycine occurred at 120 seconds and was 60 seconds long (denoted by yellow “Glutamate/glycine” in the upper left corner of the video).
(AVI)

## References

[1] Zheng JQ, Poo MM (2007). Calcium signaling in neuronal motility.. Annu Rev Cell Dev Biol.

[2] Hudmon A, Lebel E, Roy H, Sik A, Schulman H (2005). A mechanism for Ca2+/calmodulin-dependent protein kinase II clustering at synaptic and nonsynaptic sites based on self-association.. J Neurosci.

[3] Correll R, Pang C, Niedowicz D, Finlin B, Andres D (2008). The RGK family of GTP-binding proteins: regulators of voltage-dependent calcium channels and cytoskeleton remodeling.. Cell Signal.

[4] Béguin P, Nagashima K, Gonoi T, Shibasaki T, Takahashi K (2001). Regulation of Ca2+ channel expression at the cell surface by the small G-protein kir/Gem.. Nature.

[5] Finlin B, Crump S, Satin J, Andres D (2003). Regulation of voltage-gated calcium channel activity by the Rem and Rad GTPases.. Proc Natl Acad Sci U S A.

[6] Finlin B, Mosley A, Crump S, Correll R, Ozcan S (2005). Regulation of L-type Ca2+ channel activity and insulin secretion by the Rem2 GTPase.. J Biol Chem.

[7] Béguin P, Mahalakshmi R, Nagashima K, Cher D, Kuwamura N (2005). Roles of 14-3-3 and calmodulin binding in subcellular localization and function of the small G-protein Rem2.. Biochem J.

[8] Piddini E, Schmid J, de Martin R, Dotti C (2001). The Ras-like GTPase Gem is involved in cell shape remodelling and interacts with the novel kinesin-like protein KIF9.. EMBO J.

[9] Ward Y, Spinelli B, Quon M, Chen H, Ikeda S (2004). Phosphorylation of critical serine residues in Gem separates cytoskeletal reorganization from down-regulation of calcium channel activity.. Mol Cell Biol.

[10] Moyers J, Bilan P, Zhu J, Kahn C (1997). Rad and Rad-related GTPases interact with calmodulin and calmodulin-dependent protein kinase II.. J Biol Chem.

[11] Finlin B, Shao H, Kadono-Okuda K, Guo N, Andres D (2000). Rem2, a new member of the Rem/Rad/Gem/Kir family of Ras-related GTPases.. Biochem J 347 Pt.

[12] Ghiretti AE, Paradis S (2011). The GTPase Rem2 regulates synapse development and dendritic morphology.. Dev Neurobiol.

[13] Okamoto K, Narayanan R, Lee S, Murata K, Hayashi Y (2007). The role of CaMKII as an F-actin-bundling protein crucial for maintenance of dendritic spine structure.. Proc Natl Acad Sci U S A.

[14] Pi HJ, Otmakhov N, El Gaamouch F, Lemelin D, De Koninck P (2010). CaMKII control of spine size and synaptic strength: role of phosphorylation states and nonenzymatic action.. Proc Natl Acad Sci U S A.

[15] Goda Y, Colicos M (2006). Photoconductive stimulation of neurons cultured on silicon wafers.. Nat Protoc.

[16] Correll R, Botzet G, Satin J, Andres D, Finlin B (2008). Analysis of the Rem2-voltage dependant calcium channel beta subunit interaction and Rem2 interaction with phosphorylated phosphatidylinositide lipids.. Cell Signal.

[17] Van Petegem F, Chatelain F, Minor DJ (2005). Insights into voltage-gated calcium channel regulation from the structure of the CaV1.2 IQ domain-Ca2+/calmodulin complex.. Nat Struct Mol Biol.

[18] Shen K, Meyer T (1999). Dynamic control of CaMKII translocation and localization in hippocampal neurons by NMDA receptor stimulation.. Science.

[19] Bayer KU, LeBel E, McDonald GL, O’Leary H, Schulman H (2006). Transition from reversible to persistent binding of CaMKII to postsynaptic sites and NR2B.. J Neurosci.

[20] Bennett MK, Erondu NE, Kennedy MB (1983). Purification and characterization of a calmodulin-dependent protein kinase that is highly concentrated in brain.. J Biol Chem.

[21] Erondu NE, Kennedy MB (1985). Regional distribution of type II Ca2+/calmodulin-dependent protein kinase in rat brain.. J Neurosci.

[22] Hudmon A, Schulman H (2002). Neuronal CA2+/calmodulin-dependent protein kinase II: the role of structure and autoregulation in cellular function.. Annu Rev Biochem.

[23] Chang BH, Mukherji S, Soderling TR (1998). Characterization of a calmodulin kinase II inhibitor protein in brain.. Proc Natl Acad Sci U S A.

[24] Vest RS, Davies KD, O’Leary H, Port JD, Bayer KU (2007). Dual mechanism of a natural CaMKII inhibitor.. Mol Biol Cell.

[25] Crump S, Correll R, Schroder E, Lester W, Finlin B (2006). L-type calcium channel alpha-subunit and protein kinase inhibitors modulate Rem-mediated regulation of current.. Am J Physiol Heart Circ Physiol.

[26] Yada H, Murata M, Shimoda K, Yuasa S, Kawaguchi H (2007). Dominant negative suppression of Rad leads to QT prolongation and causes ventricular arrhythmias via modulation of L-type Ca2+ channels in the heart.. Circ Res.

[27] Fan M, Buraei Z, Luo H, Levenson-Palmer R, Yang J (2010). Direct inhibition of P/Q-type voltage-gated Ca2+ channels by Gem does not require a direct Gem/Cav{beta} interaction.. Proc Natl Acad Sci U S A.

[28] Wang G, Zhu X, Xie W, Han P, Li K (2010). Rad as a novel regulator of excitation-contraction coupling and beta-adrenergic signaling in heart.. Circ Res.

[29] Flynn R, Zamponi GW (2010). Regulation of calcium channels by RGK proteins.. Channels (Austin).

[30] Merrill M, Chen Y, Strack S, Hell J (2005). Activity-driven postsynaptic translocation of CaMKII.. Trends Pharmacol Sci.

[31] Yamauchi T (2005). Neuronal Ca2+/calmodulin-dependent protein kinase II–discovery, progress in a quarter of a century, and perspective: implication for learning and memory.. Biol Pharm Bull.

[32] Bilan P, Moyers J, Kahn C (1998). The ras-related protein rad associates with the cytoskeleton in a non-lipid-dependent manner.. Exp Cell Res.

[33] Pan J, Fieles W, White A, Egerton M, Silberstein D (2000). Ges, A human GTPase of the Rad/Gem/Kir family, promotes endothelial cell sprouting and cytoskeleton reorganization.. J Cell Biol.

[34] Leone A, Mitsiades N, Ward Y, Spinelli B, Poulaki V (2001). The Gem GTP-binding protein promotes morphological differentiation in neuroblastoma.. Oncogene.

[35] Ward Y, Yap S, Ravichandran V, Matsumura F, Ito M (2002). The GTP binding proteins Gem and Rad are negative regulators of the Rho-Rho kinase pathway.. J Cell Biol.

[36] Aresta S, de Tand-Heim M, Béranger F, de Gunzburg J (2002). A novel Rho GTPase-activating-protein interacts with Gem, a member of the Ras superfamily of GTPases.. Biochem J.

[37] Oyama F, Kotliarova S, Harada A, Ito M, Miyazaki H (2004). Gem GTPase and tau: morphological changes induced by gem GTPase in cho cells are antagonized by tau.. J Biol Chem.

[38] Hatzoglou A, Ader I, Splingard A, Flanders J, Saade E (2007). Gem associates with Ezrin and acts via the Rho-GAP protein Gmip to down-regulate the Rho pathway.. Mol Biol Cell.

[39] Paradis S, Harrar D, Lin Y, Koon A, Hauser J (2007). An RNAi-based approach identifies molecules required for glutamatergic and GABAergic synapse development.. Neuron.

[40] Asrican B, Lisman J, Otmakhov N (2007). Synaptic strength of individual spines correlates with bound Ca2+-calmodulin-dependent kinase II.. J Neurosci.

[41] Jourdain P, Fukunaga K, Muller D (2003). Calcium/calmodulin-dependent protein kinase II contributes to activity-dependent filopodia growth and spine formation.. J Neurosci.

[42] Sanabria H, Swulius M, Kolodziej S, Liu J, Waxham M (2009). {beta}CaMKII regulates actin assembly and structure.. J Biol Chem.

[43] Gutiérrez RC, Flynn R, Hung J, Kertesz AC, Sullivan A (2009). Activity-driven mobilization of post-synaptic proteins.. Eur J Neurosci.

[44] Li Q, Lau A, Morris T, Guo L, Fordyce C (2004). A syntaxin 1, Galpha(o), and N-type calcium channel complex at a presynaptic nerve terminal: analysis by quantitative immunocolocalization.. J Neurosci.

